# A Combinatory Antibody–Antigen Microarray Assay for High-Content Screening of Single-Chain Fragment Variable Clones from Recombinant Libraries

**DOI:** 10.1371/journal.pone.0168761

**Published:** 2016-12-21

**Authors:** Nina Persson, Bo Jansson, Nicolai Stuhr-Hansen, András Kovács, Charlotte Welinder, Lena Danielsson, Ola Blixt

**Affiliations:** 1 Chemical Glyco-Biology Laboratory, Department of Chemistry, Copenhagen University, Copenhagen, Denmark; 2 Division of Oncology and Pathology, Department of Clinical Sciences Lund, Lund University, Lund, Sweden; 3 Division of Clinical Chemistry and Pharmacology, Department of Laboratory Medicine, Lund University, Lund, Sweden; 4 Centre of Excellence in Biological and Medical Mass Spectrometry (CEBMMS), Biomedical Centre D13, Lund University, Lund, Sweden; National Cancer Institute at Frederick, UNITED STATES

## Abstract

We have developed a combinatory antibody–antigen microarray for direct screening of multiple single-chain fragment variable (scFv) clones with no need for pre-purification or enrichment before screening. The straightforward workflow allows for early selection of binders to predefined peptide and glycopeptide targets. A capture antibody is contact printed on microarray slides, side by side with the antigens of interest. A large number of scFv clones, in supernatants, are printed on top of the capture antibody and the antigen in a “spot-on-spot” print. The printed scFv clones, which bind to the capture antibody, are detected using biotinylated antigen, while the binding of scFv clones to the printed antigen is detected through a mouse anti-tag antibody. Two different analyses are thus performed on the same slide, generating two kinds of information: one on the ability of an individual scFv clone to bind to the soluble form of the antigen, which may favour selection for higher affinity rather than avidity, while the other allows the identification of large numbers of clones, simultaneously, due to the binding of scFv clones to densely presented antigens, thus providing an overall increased hit rate. The functionality of the new screening approach was illustrated through the generation of antibodies against peptides from the chaperone complex Ku70/Ku80 and the GalNAcα-serine/threonine epitope on the IgA1 alpha chain hinge region. In total, 659 scFv clones were screened with a hit rate of approximately 20%. This approach allowed the identification of functional antibodies in both cases, illustrating the usefulness and capacity of this combinatory microarray screening technique for efficient analysis and validation of antibodies at an early stage of antibody generation.

## Introduction

The use of antibodies in therapeutic approaches is very promising, as demonstrated by the rising number of antibody-based drugs approved by the US Food and Drug Administration [1]. Many steps in the generation of new antibodies have been optimized and automated [2]. However, finding new targets and developing next generation antibody-based therapeutics are still challenges that needs to be addressed [3].

Regardless of the origin of an antibody library, it is important to find ways of efficiently selecting, screening, and identifying the most useful antibodies for a specific purpose. In the screening of soluble antibodies, methods such as bead-based flow cytometry [4, 5], microarrays [6, 7] and label-free array-based biosensors [8] have been developed, which have enhanced the throughput of screening. These methods have attractive features such as multiplex presentation of antigens on beads and the minute amount of antigen required. Moreover, they have the capacity for multi-testing of target structures and the analysis of a large number of clones. One way to achieve high quality screening would be to design a platform that generates more information on the characteristics of the antibody at an early stage, using only small amounts of antigen (in the range of 5–50 μg). It would also be advantageous if this platform could be used in an automated system to allow the analysis of a large number of clones.

Efforts have been made to improve the throughput and quality of the output of the microarray method. For example, Babel et al. [9] were able to analyse 192 scFv clones in 3–4 days using direct printing of purified scFv clones and detecting the target protein in solution. It has also been shown that it is possible to immobilize purified scFv clones through an anti-tag antibody in spot-on-spot printing [7].

In the present study we isolate new scFv clones, using the microarray technology for efficient specificity evaluation. We have developed a high-content screening method for scFv clones, using a combinatory microarray approach where the antibodies could be simultaneously screened on a combined antibody–antigen microarray. Such a procedure would allow early identification of different types of binders. We are using non-purified scFv clones in supernatant as a detector and a capture antibody of a small, defined, modified peptide epitope using spot-on-spot printing. This could allow the high-affinity binders for pre-defined peptides and post-translationally modified epitopes to be selected in a high-throughput protocol. To illustrate the capacity and usefulness of this combinatory microarray screening method, we present and evaluate data from experiments with scFv clones against three different potentially therapeutic targets, the nucleus-associated proteins Ku70/80 complex (consisting of X-ray repair cross-complementing proteins 6 and 5 respectively), also exposed on the plasma membrane of the majority of tumours [10] and to the O-linked GalNAcα-Ser/Thr epitope (Tn antigen) found on a variety of cancer-associated proteins such as the IgA1 alpha chain hinge region [11], MUC-1 and MUC5Ac [12, 13].

## Material and Methods

### Peptide synthesis and protein conjugates

The peptides and glycopeptides used in the study are given in Table 1. They were prepared by automated peptide synthesis on a Syro II peptide synthesizer (MultiSynTech, Witten, Germany) using a modified 9-fluorenylmethoxycarbonyl–solid-phase peptide synthesis method, as described previously [14, 15]. All HPLC-purified products were identified by LC-MS and isolated at higher than 90% purity. A 10 mM solution of each peptide in water was prepared for keyhole limpet hemocyanin (KLH, Pierce) and bovine serum albumin (BSA) conjugation using the glutaraldehyde method [16]. BSA (20mg) was dialysed against phosphate-buffered saline (PBS) overnight, generating a 10 mg/mL dialysed solution. KLH was solubilized in 2.0 mL Milli-Q® water (10 mg/mL). KLH solution (200 μL) was mixed with 20 μL (10 mg/mL) or 40 μL of a solution containing selections of the peptides **1–10** (5 mg/mL, Table 1). BSA (200 μL) was mixed with 10 μL (10 mg/mL) or 20 μL of the 5 mg/mL peptide solution and incubated for 30 min at room temperature (RT). Glutaraldehyde (200 μL, 2% freshly prepared solution in Milli-Q water) was added, and the vials were gently rotated for 1 hour at RT. All conjugates were dialysed against PBS overnight using a Slide-A-Lyzer device with 10 kDa MW cut-off membranes. The conjugates were removed and the membranes were washed with one volume of PBS. The conjugates were then lyophilized in a 10 mL plastic tube and later used for immunization. MALDI-TOF analysis of the BSA conjugates tested (peptides 1, 2, 6 and 7) indicated an average peptide incorporation of five per BSA molecule (S2 Table).

**Table 1:** Amino acid sequence of peptides used in the antibody generation process.

Peptide no.	Protein	Sequence
Peptide **1**	Ku70 subunit	^409^YFVALVPQEEELDDQKIQVT
Peptide **2**	Ku70 subunit	^164^KRIMLFTNEDNPHGNDSAKA
Peptide **3**	Ku70 subunit	^501^EQAVDLTLPKVEAMNKRLGS
Peptide **4**	Ku70 subunit	^521^LVDEFKELVYPPDYNPEGKV
Peptide **5**	Ku80 subunit	^610^EEASNQLINHIEQFLDTNETP
Peptide **6**	Ku80 subunit	^681^DGITLITKEEASGSSVTAEEAKKFL
Peptide **7**	Ku80 subunit	^641^AFREEAIKFSEEQRFNNFLK
Peptide **8**	IgA1 hinge region	^103^VPSTPPTPSPSTPPTPSPSA
Peptide **9**	IgA1 hinge region	^103^VPSTPPTPSPSTPPTPSPSA
Peptide **10**	IgA1 hinge region	^103^VPSTPPTPSPSTPPTPSPSA
Peptide **11**	IgA1 hinge region	^103^VPSTPPTPSPSTPPTPSPSA
Peptide **12**	Control peptide	SGSGTLYVGK

Underlined amino acids indicate O-glycosylation with GalNAc.

### Antibody production

#### Ethics

Immunizations were done in accordance with animal law and EU directive 2010/63/EU including a general ethical permit for immunization experiments

#### Immunization and antibody library construction

Balb/c mice were immunized at Innovagen AB, Lund, with 175 μg of each peptide conjugate of the heterodimeric Ku70/80 protein complex (peptides **1–4** from the Ku70 subunit and peptides **5–7** from the Ku80 subunit), or glycopeptides of the IgA1 hinge region with one or several GalNAcα-Ser/Thr epitopes (peptides **8–10**) conjugated to KLH using the glutaraldehyde method. This was followed by a booster dose of 175 μg peptide conjugate after 27 days. The mice were sacrificed 14 days after the booster dose and the spleens were stored in RNAlater® solution (Qiagen) at -20°C until the RNA was collected with a Fastprep Cell Disrupter FP120 (Q-Biogene) and the RNeasy Plus Mini kit (Qiagen). cDNA synthesis was performed using 1 μg RNA with random hexamer primers and ThermoScript Reverse Transcriptase (Invitrogen). Specific antibody genes were isolated using PCR and cloned into the phage display vector pAK100 (kindly provided by Prof. A. Plückthun, University of Zurich, Switzerland) [17, 18], using the primers and conditions described by Schaefer et al. [19], with a slight modification. The outer primers were 5′-phosphorylated to allow an extra rolling-circle amplification step to improve the restriction enzyme cutting of the antibody-fragment-encoding genes [20]. The libraries generated consisted of 1–2 x10^7^members, according to standard titration. The scFv-displaying phages were rescued using VCSM13 helper phages [17].

#### Selection of scFv antibodies from the phage library

All centrifugation steps of bacteria were performed at 3000 x g and the culture medium used throughout was LB medium supplemented with 25 μg/mL chloramphenicol and 15 μg/mL tetracycline, unless otherwise stated. The phage libraries were subjected to three rounds of selection using magnetic streptavidin beads (M280, Invitrogen) and Immuno Tubes (Thermo Scientific). Biotinylated antigen (50–100 nM) in 0.05% Tween 20 in PBS (TPBS) with 3% (w/v) BSA was incubated with 100 μL streptavidin beads for 1 hour at RT or overnight at 4°C. The beads were washed twice with 3% BSA in TPBS before blocking with 5% BSA in TPBS for 1 hour at RT. The phage library was pre-selected with biotinylated non-target peptide before selection with the biotinylated target peptides for 2 hours at RT or overnight at 4°C, followed by washing three times each in 3% BSA in TPBS, TPBS and PBS. Bound phages were eluted with 1 mg/mL trypsin (Sigma Aldrich) in PBS for 30 min at RT before adding 2 mg/mL of the trypsin inhibitor aprotinin (Roche) to the supernatant, and the beads were washed once in PBS. Eluted phages were allowed to infect exponentially growing *E*. *coli* XL1-Blue competent cells (Agilent) for 30 min, under slow rotation (130 rpm) at 37°C. Infected bacteria were plated on LB agar-plates containing 1% glucose, 15 μg/mL tetracycline and 25 μg/mL chloramphenicol, followed by incubation overnight at 30°C. Bacterial colonies on the plates were resuspended in 10 mL culture medium, centrifuged, and the pellet was resuspended in 1 mL culture medium, after which 50% glycerol was added to make a glycerol stock solution. A new phage stock solution was made by inoculating 10 mL culture medium with 20–40 μL of the glycerol stock solution, which was incubated at 37°C, with 250 rpm rotation until an optical density (OD_600_) of 0.5 was reached. The helper phages (VCSM13, 6 x 10^9^ plaque-forming units (pfu)/mL) were allowed to infect the culture, which was then incubated for 30 min at 37°C under rotation at 50 rpm. Expression was thereafter induced by the addition of isopropyl β-D-1-thiogalactopyranoside (IPTG) to a final concentration of 100 μM. The cell culture was then incubated at 25°C, 200 rpm, overnight. The culture was centrifuged, and the phages were precipitated from the supernatant by adding 1⁄4 volume of 20% PEG6000 (BDH Biochemicals) with 2.5 M NaCl, followed by centrifugation at 4800 x g for 30 min at 4°C. The phage pellet was resuspended in PBS and used in the next round of panning, which was performed on Immuno Tubes coated with peptide-BSA conjugate, or non-target peptide-BSA conjugate for preselection, in coating buffer (0.1 M NaCO_3_, pH 9.1). The third panning stage was carried out with 5 or 50 nM biotinylated peptides on streptavidin-coated beads. The phage solutions from final round of selection were analysed with enzyme-linked immunosorbent assay (ELISA) and/or phage-binding microarray assay.

#### Expression and purification of soluble scFv antibodies

After three rounds of selection, the polyclonal scFv gene fragments were subcloned using SfiI enzyme (New England Biolabs) into the expression vector pJB33 (kindly provided by Prof. A. Plückthun, University of Zurich, Switzerland) [19]. The resulting constructs were transformed into *E*. *coli* XL1-Blue competent cells and grown overnight on LB agar-plates with 25 μg/mL chloramphenicol and 1% glucose at 37°C. Individual colonies were picked and grown in LB-medium containing 25 μg/mL chloramphenicol and 1% glucose, overnight at 37°C, in 96-well plates. The overnight cultures were inoculated into fresh LB-medium containing 25 μg/mL chloramphenicol, and grown for 3.5 hours before induction with IPTG (final concentration 0.5 mM) and cultivation overnight at 37°C, 130 rpm. Bacterial cells were pelleted by centrifugation and the supernatants were used for screening in the combinatory microarray screening assay.

ScFv clones chosen after the combinatory microarray screening were grown overnight in LB medium containing 25 μg/mL chloramphenicol and 1% glucose, at 37°C and 200 rpm. Part of the overnight culture was added to Terrific Broth medium containing 25 μg/mL chloramphenicol, cultured until the OD_600_ reached 0.5, and expression was then induced with 1 mM of IPTG, after which the culture was incubated overnight at 37°C, 200 rpm. Bacterial cells were centrifuged and the pellet was resuspended in 1 mg/mL lysozyme (Sigma Aldrich) in sucrose solution (20% sucrose (w/v), and 1 mM EDTA in 30 mM Tris buffer, pH 8.0), followed by agitation on ice for 1 hour. The solution was centrifuged for 3000 x g for 30 min at RT, and the supernatant was sterilized by 0.2 μm filtration before the scFv clones were purified on a Ni-NTA agarose column. The protein concentration was estimated using a NanoDrop spectrophotometer 2000 (Thermo Scientific) and the purity was evaluated using SDS-PAGE.

#### ELISA

Maxisorp plates, 96-well (Nunc, Thermo Scientific) were pre-coated with 1 μg/mL streptavidin (Sigma Aldrich) in 0.1 M NaCO_3_ buffer, pH 9.1, and washed three times in TPBS. Biotinylated peptides **7** and **12** (2 μg/mL) were added to the streptavidin-coated surface. The wells were washed three times with TPBS and then blocked with 3% BSA in TPBS for 1 hour. Titrated phages (serial diluted from 1:4 to 1:512) were added to the plate and incubated for 1.5 hours at RT. After three TPBS washes the binding phages were detected with anti-M13 monoclonal mouse antibodies conjugated to horseradish peroxidase (HRP) (GE Healthcare Life Sciences, 27942101) diluted 1:1000 in TPBS. The plates were washed three times in TPBS and then 1 mg/mL o-phenylenediamine (Arcros Organics) dissolved in 0.1 M citric acid–phosphate buffer supplemented with 0.012% H_2_O_2_ was used to develop a signal. The reaction was stopped with 1 M HCl, and the plate was read at 490 nm in a Varioskan Flash ELISA reader (Thermo Scientific).

### Fabrication of the microarray for phage-binding analysis

#### Covalent immobilization of peptides/glycopeptides

Peptides/glycopeptides (100 μM) solubilized in print buffer (300 mM phosphate buffer, pH 8.5, containing 0.005% Tween-20) were printed by robotic pin deposition using a MicroGrid II arrayer (BioRobotics, Genomics Solutions, 60 nL/deposit, quilled pins, 250 μm pitch) onto N-hydroxysuccinimide (NHS)-activated glass array slides (SCHOTT NEXTERION® Slide H) [14, 15]. The printed slides were placed in a high-humidity chamber for 1 hour. Remaining NHS groups were blocked by immersion in NHS blocking buffer (50 mM ethanolamine in 50 mM borate buffer, pH 9.2) for 30 min just before use.

#### Phage-binding microarray assay

Slides with covalent immobilized peptides were rinsed three times with distilled water, and spin dried in a Galaxy Miniarray centrifuge (VWR) before incubation in the phage stock solution diluted 1:2 or serial dilution (from 1:2 to 1:2000) in PLI-P buffer (6.5 mM Na_2_HPO_4_, 1.5 mM KH_2_PO_4_, pH 7.4 containing 500 mM NaCl, 3 mM KCl, 1% BSA and 1% Triton X-100) for 1.5 hours at RT with agitation. After three washing steps in PBS, anti-M13 monoclonal mouse antibodies (GE Healthcare Life Sciences, Cat. No 27-9420-01), diluted 1:100 (10 μg/mL), were added and incubated for 1 hour at RT with gentle agitation. After three washing steps, Cy5-conjugated goat anti-mouse IgG (H+L) (Jackson ImmunoResearch Labs, Cat. no 115-175-146) diluted 1:500 and incubated for 1 hour at RT, was used to detect binding. After a final washing step, the slides were quickly rinsed in distilled water and air dried. The fluorescence signal was measured using a ScanArray 5000 (PerkinElmer) confocal scanner.

### Printing of the combinatorial microarray

#### Covalent immobilization of anti-His-antibody and antigens

Anti-His-antibody (1 mg/mL, R and D Systems, Cat. no MAB050) and/or antigens (100 μM) solubilized in print buffer (300 mM phosphate buffer, pH 8.5, containing 0.005% Tween-20) were printed by robotic pin deposition, as described above, onto NHS-activated glass array slides. The printed slides were then placed in a high-humidity chamber for 1 hour as recommended by manufacturer (SCHOTT NEXTERION® Slide H). Remaining NHS groups were blocked by immersion in NHS blocking buffer, as described above.

#### Combinatory spot-on-spot printing

Slides with both covalently immobilized anti-His antibody and peptides were rinsed three times in PBS/distilled water and placed in the printer in the same position as for the first print. Spot-on-spot printing was then carried out with the supernatant from freshly produced scFv clones. Each scFv clone was printed on top of the anti-His antibody and on top of each immobilized antigen. Printing on each spot was repeated three times, followed by overnight incubation at 4°C in a high-humidity chamber. The slides were then rinsed three times in PBS.

### Combinatorial microarray screening assays

#### Capture assay

Biotinylated peptides/glycopeptides (10–100 μg/mL) in PBS were incubated on printed slides for 1 hour at RT with gentle agitation. After three washes in PBS, binding was detected with streptavidin-Alexa Fluor 647 (ThermoFisher Scientific, S32357), diluted 1:1000 in PBS, for 1 hour at RT. After the final washing step, the slides were rinsed with distilled water and air dried. The fluorescence was measured using a ScanArray 5000 confocal scanner, as described above.

#### Antigen assay

After scanning, the slides from the capture assay were incubated with mouse anti-His antibody (3 μg/mL) for 1 hour and washed three times in PBS, followed by detection with the secondary antibody, Cy3-conjugated goat anti-mouse IgG (H+L) (Jackson ImmunoResearch Labs, Cat. no 115-165-003) diluted 1:500 in PBS, and incubated for 1 hour at RT. All incubation steps were separated by three washing steps in PBS. After the final washing step, the slides were rinsed in water and air dried. Fluorescence was measured detected as described above.

#### Flow cytometry and image stream analysis

The leukemia cell line, Jurkat (ATCC TIB-152), and prostate cancer cell line, LnCap (ATCC CRL-1740), were obtained from the ATCC (Rockville, MD, USA). Both cell lines were grown under standard cultivation conditions, 37°C, 5% CO_2_ in RPMI1640 medium supplemented with 10% fetal bovine serum. The cells were incubated with scFv clone (H5 or G2-H7), anti-Ku80 mouse antibody 5C5 (Abcam, Cat. no ab119935), anti-Tn antigen antibody GOD3-2C4 [21], non-binding scFv clone or mouse IgG1 as negative controls. The scFv clones were detected with a mouse anti-His antibody, and polyclonal allophycocyanin-conjugated anti-mouse IgG antibody (Jackson ImmunoResearch Labs, Cat. no 115-136-146). The scFv clone H5 targeting peptide 6 was analysed with flow cytometry using a BD FACSAria II, cell sorter (BD Biosciences), using Sytox® Green nucleic acid stain (Invitrogen) as viability stain. The scFv clone G2-H7 was analysed with an ImageStream®x Mark II flow cytometer (Merck).

## Results

We have designed and incorporated a combinatory microarray screening assay into the antibody generation process for the efficient identification and early evaluation of useful antibodies. The crucial target preparation stage, using solid-phase peptide synthesis together with isolation of antibody genes in libraries in phage display format, is followed by selection and evaluation of phage display antibodies in bulk, using the microarray platform. Primary screening of soluble single antibody clones is then performed using the combinatory microarray screening assay, after which selected clones are further evaluated using a biophysical analysis method.

### Evaluation of phage selection using the phage-binding microarray assay

**Fig 1:**
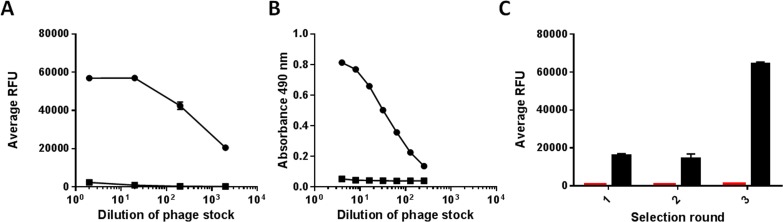
Phage pool evaluation using phage stock solution from selection with Ku80 peptide 6. **A**) Titration curve for phages (from third round of selection) binding to Ku80 peptide 6 (■) and peptide **7** (●) in the phage binding (assay average value of triplicates). Detection was possible down to 1/2000 dilution. **B**) Titration curve for phages (from third round of selection) binding to Ku80 peptide **7** (●) and the negative control, peptide **12** (■) in ELISA (single value). Detection was possible at 1/284 dilution. **C**) Analysis of phage binding to a representative peptide (black bar) and negative control peptide (red bar) after selection rounds 1, 2 and 3 using the phage-binding assay.

Antibody scFv libraries, each containing approximately 10^7^ different members, were constructed from the spleens of immunized mice and displayed on phages. Each library was subjected to three rounds of selections on streptavidin-coated beads and Immuno Tubes using selected antigens. After each selection round, phages were eluted, amplified and evaluated against 14 different peptide epitopes in the phage-binding microarray assay. This allowed fast-track analysis against multiple epitopes. Phage stock solution from the final selection round against Ku80 peptide **7** was titrated and analysed with the phage binding microarray assay (Fig 1A) using 14 printed peptides/glycopeptides: peptides **5–9** and peptide **11** (Table 1), and peptides **13–20** (S1 Table), and ELISA phage-binding assays (Fig 1B) using two peptides, **7** and **12** (Table 1). A 1:10 dilution series was used for the phage-binding microarray assay, while a 1:2 dilution series was used in the ELISA phage-binding assay. For example, the results for phage binding to Ku80 peptide **7** (Fig 1A and 1B) showed that a more diluted phage pool could be used in the phage-binding microarray assay than in the ELISA phage-binding assay (1:2000 versus 1:128 dilution of the phage stock solution). The remaining 13 peptides, represented by peptide **6** in Fig 1A, were negative in the phage-binding microarray assay, as was the negative control, peptide **12**, in the ELISA phage-binding assay (Fig 1B). This shows that the phage-binding microarray assay is more sensitive than the phage-binding ELISA assay, and is useful in evaluating each round of selection against multiple peptides/glycopeptides (Fig 1C) in a high-throughput manner.

### High-content combinatory microarray screening of multiple scFv clones

**Fig 2:**
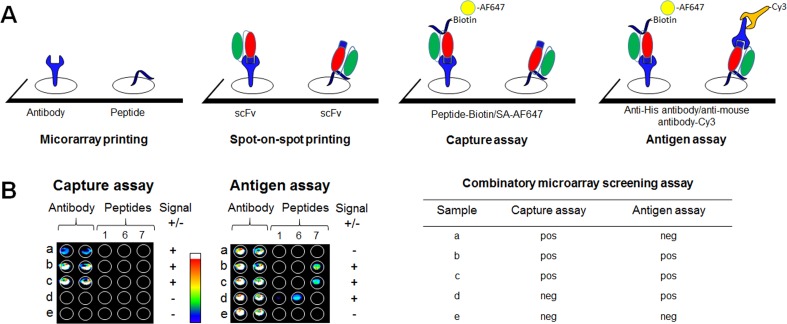
The combinatory microarray screening assay. **A**) Capture antibody and peptides are printed on a microarray slide before adding scFv in a spot-on-spot print. Subsequent detection of captured biotinylated peptide with streptavidin-conjugated fluorophore (SA-AF647), and bound scFv clone with anti-His and labelled anti-mouse antibodies. **B**) Analysis of five scFv antibodies. In the capture assay, three clones (a-c) are identified as positive binders to the biotinylated Ku80 peptide **7**, while d and e are negative. Two scFv clones (b and c) were identified as Ku80 peptide **7** binders) and one scFv clone (*d*) was bound to Ku80 peptide **6**. One scFv clone (*e*) was negative for all tested peptides. The table summary of the combinatory microarray screening assay shows the four possible outcomes of screening.

Phages with significant binding to target peptides/glycopeptides in the phage-binding microarray assay were converted with a batch procedure to soluble scFv clones and produced in bacteria as single clones. To increase the speed and selection of scFv binders with the desirable specificities and high affinities, we designed a microarray screening method in which all the selected scFv clones could be evaluated simultaneously on a single slide, without the need of scFv purification. A schematic workflow of the assay is presented in Fig 2A. The capture antibody (anti-His antibody) and the peptides are printed individually on a microarray slide before adding scFv clones by spot-on-spot printing. In the capture assay, scFv clone capture by the anti-His antibody and binding to biotinylated peptide are detected with streptavidin- Alexa Fluor 647. The same slide is then used in the antigen assay, where binding to immobilized peptide is detected with the anti-His tag antibody and an anti-mouse antibody conjugated fluorophore. In a representative experiment, fresh supernatants from individual *E*. *coli* clones expressing scFv from selection on Ku70 peptide (peptide **1**) and Ku80 peptides (peptides **6** and **7**) were printed directly via spot-on-spot printing on the anti-His tag capture antibody and on the immobilized peptide antigens (**1**, **6**, and **7**), and were then analysed in the capture assay and the antigen assay. In the selected area of the capture assay shown in Fig 2B (the whole area of the array is shown in S1 Fig) we identified three captured scFv clones (a, b and c) binding to Ku80 peptide **7** in solution, while two scFv clones (d and e) did not bind to Ku80 peptide **7** in solution. When studying the same clones in the antigen assay (Fig 2B) two scFv clones (b and c) were found to be Ku80 peptide **7** positive and one scFv clone (d) Ku80-peptide **6** positive, while the remaining scFv clones did not bind to the immobilized antigens. None of the scFv clones was cross-reactive with the other printed Ku70/80 peptides and the secondary antibody bound to the printed capture antibody, as indicated by the double spots of the anti-His tag antibody in samples a-e in Fig 2B. The combinatory microarray screening assay resulted in four outcomes: 1) a positive signal in the capture assay, together with a negative signal in the antigen assay (e.g. sample a); 2) positive signals in both the capture assay and the antigen assay (e.g. samples b, c); 3) a negative signal in capture assay together with a positive signal in the antigen assay (e.g. sample d); and 4) negative signals in both assays (e.g. sample e).

The combinatory microarray screening assay was also used in the screening of scFv clones selected on glycopeptides **8** and **9** using a second phage library. None of the scFv clones could bind to the biotinylated antigen presented in solution in the capture array. However, several scFv clones bound to glycopeptide **9** in the antigen array (S2 Fig). This demonstrates that the combinatory microarray screening assay is also useful for detecting scFv clones that target glycopeptides.

Using this combinatory microarray screening assay, 93 of 224 scFv clones targeting Ku80 peptide **7** were identified; 71 clones were identified in the capture array and 75 in the antigen array (Table 2). Thirty-one of 111 scFv clones targeting glycopeptide **9** could be identified in the antigen array, but none was detected in the capture array (Table 2). Cross-reactivity to other peptides/glycopeptides in a first screening assay was estimated to about 3%.

**Table 2:** Results of scFv screening using the combinatory microarray screening assay, in terms of the number of peptide-positive clones and the total number of clones.

Targets in selection	Capture array	Antigen array	Combinatory array
Ku80 peptide (**7**)	71/224	75/224	93/224
Ku70/Ku80 peptide (**1**,**6**)	4/324	15/324	16/324
Glycopeptide (**9**)	0/111	31/111	31/111

### Evaluation of the combinatory microarray screening assay

**Fig 3:**
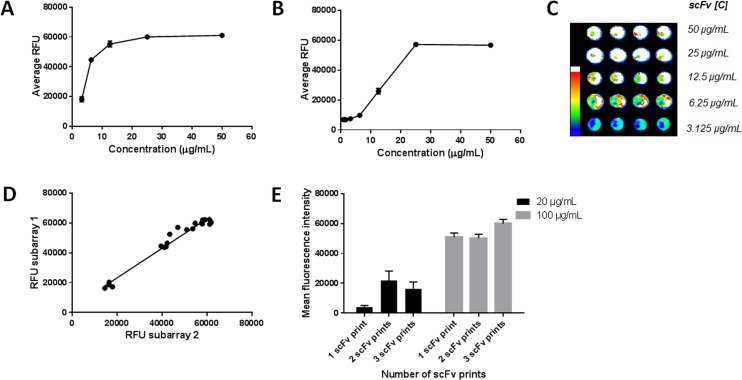
Evaluation of the combinatory microarray screening assay. **A**) Titration curve for purified scFv H5, binding to biotinylated Ku80 peptide **7** (100 μg/mL). **B**) Titration curve for purified scFv G2-H7, binding to biotinylated glycopeptide **9** (100 μg/mL). **C**) Signal intensity with different scFv H5 concentrations in the capture assay. **D**) Subarray-to-subarray reproducibility of the capture assay. ScFv H5 is printed on top of the capture antibody, and binding to biotinylated Ku80 peptide **7** is detected (100 μg/mL). **E**) Evaluation of spot-on-spot printing. The scFv clone G2-H7 (20 μg/mL and 100 μg/mL) was printed once, twice or three times in spot-on-spot printing on the capture anti-His tag antibody (1 mg/mL).

Selected scFv clones were produced on a larger scale, purified, and used to further evaluate the robustness of the combinatory microarray screening assay. The titration curve of scFv H5 binding to Ku80 peptide **7** in solution showed that this clone could be detected down to a concentration of 3 μg/mL, while the detection level for the G2-H7 clone, binding to glycopeptide **9**, was 12.5 μg/mL (Fig 3A and Fig 3B, respectively). The signal intensity of printed scFv H5 on capture antibody and detecting target peptide in solution is shown in Fig 3C. The scFv H5 was used to analyse the array-to-array variation by printing 4 spots of each concentration, on two different subarrays in the capture assay. The correlation between the two subarrays was good, giving an R^2^ value of 0.97 (Fig 3D). Thus, the combinatory microarray screening assay was reproducible. To evaluate the positional accuracy of the spot-on-spot printing, the scFv G2-H7 was printed once, twice or three times on the capture antibody (anti-His antibody). An increase in the fluorescence signal was seen between 1 print and 2–3 prints for concentrations of both 20 μg/mL and 100 μg/mL, showing that the scFv is indeed printed on the same spot (Fig 3E)

### Functionality of the selected clones

**Fig 4:**
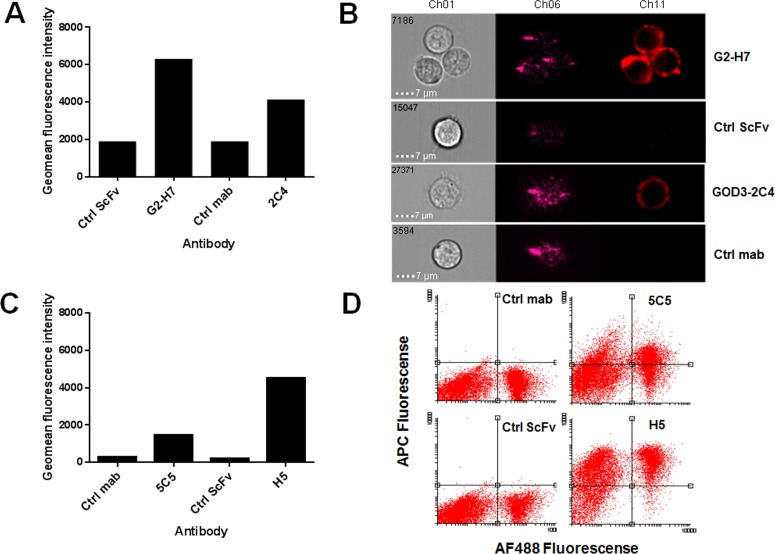
Flow cytometry evaluation of clones from the combinatory microarray screening assay. Tn-positive Jurkat cells (A-B) were stained with anti-Tn scFv G2-H7, GOD3-2C4 (positive control) and a non-binding scFv clone or mouse Ig (negative controls), and analysed with image stream flow cytometry. **A**) Geomean of APC fluorescence signal from the secondary antibody. **B**) Three individual representative pictures of stained cells from image stream flow cytometry analysis, showing (Ch01 = bright field, Ch06 = side scatter, Ch11 = APC) cells or clusters of cells with characteristic cell surface staining. The cell line LnCap (**C**-**D**) was stained for viability with Sytox® Green (AF488) and with scFv H5 and control antibodies. **C**) Geomean of APC fluorescence signal from secondary antibody of viable cells (AF488 negative). **D**) Cell-sized objects were gated with forward and side scatter and analysed for APC and AF488 fluorescence.

One of the scFv clones binding to Ku80 peptide **7** and one scFv clone binding to glycopeptide **9** were chosen for the second line of testing using flow cytometry, to determine their functionality on biological samples and possible use as biomarkers. The scFv clone G2-H7 (targeting glycopeptide **9**) was evaluated with image stream flow cytometry using a known Tn-positive Jurkat cell line [22]. The geomean fluorescence intensity of allophycocyanin (APC) labelling of the secondary antibody clearly shows that the scFv G2-H7 clone binds to Jurkat cells (Fig 4A). When comparing scFv G2-H7, GOD3-2C4 (positive control) and a non-binding scFv (negative control) on individual cells using image stream flow cytometry, a very similar binding pattern of surface staining on the anti-Tn antibodies was seen (Fig 4B). The Ku80 scFv H5 clone was tested against the LnCap cell line, a Ku70/80 plasma-membrane-positive cell line, using flow cytometry cells were stained for viability with Sytox® Green (Fig 4C and 4D). The geomean of antibody binding (H5) to viable cells was compared to the binding of a commercial antibody (5C5) (Fig 4C). The two antibodies bound to both viable and dead cells (Fig 4D).

## Discussion

Many different techniques, from ELISA to flow cytometry, using cells, full length protein or small peptides, have been described in the selection and screening of phage libraries for the generation of new antibodies [2]. The selection assay should be able to differentiate between targeted and non-targeted binding phages, while a screening assay should accurately identify individual clones in an automated fashion with high specificity [2, 23]. In this study, small peptide- and glycopeptide-based targets were chosen to select binders for a specific epitope. Using a defined target epitope throughout the selection and screening of scFv clones and combining this with efficient synthesis and microarray display of target epitopes, we increase the probability of detecting specific and non-specific binders at an early stage, can perform comprehensive epitope characterization of the scFv antibodies generated and successfully generate panels of defined scFv antibodies in a short time frame.

In the phage-binding assay, using the microarray format, we were able to evaluate each round of selection on multiple targets, using both similar and very different epitopes, at the same time. The phage-binding assay is thus an important tool in the evaluation of selection of phages, making it possible to identify binders or cross-reactive binders in the phage pool without having to test individual phage clones at an early stage. This time-saving strategy allows the evaluation of each individual selection step and makes it possible to follow the selection more accurately, thereby constituting a valuable tool in the decision regarding which selection to continue with for the production of soluble scFv clones.

The microarray technique has been used for many applications, for example, glycopeptide arrays for the analysis of autoantibodies [13], glycopeptide array for the analysis of vaccine [24] or antibody microarrays for screening of fusion antibodies [25]. Being able to obtain information on the binding characteristics of scFv clones, at an early stage, will lead to savings in both time and cost in the production and development of antibodies. Kibat et al. [25] demonstrated the utility of microarrays in the selection of scFv clones by making a fusion protein with a human IgG1 Fc part, using direct printing of the purified antibody and incubation with clinical samples. Angenendt et al. [26] screened scFv supernatants against printed target protein in spot-on-spot arrays demonstrating a higher-throughput assay.

Our combinatory microarray screening assay has increased the information available on the scFv clones by presenting the target in two forms: immobilized on a solid surface and in solution, which increases the probability of finding antibodies with different characteristics. Two different analyses are thus performed on the same slide, generating two kinds of information: one on the ability of the individual scFv clone to bind to the soluble form of the antigen, favouring selection for higher affinity rather than avidity, and the other on the binding of scFv to a densely presented antigen, enabling the identification of clones with lower affinity, and giving a higher overall hit rate.

In this combinatory microarray screening assay, there is a theoretical possibility that the biotinylated peptides added in the capture assay could elute scFv clones from the printed peptides in the antigen assay. However, in most cases, there will not be a sufficient concentration of peptides in the solution to elute scFv clones from the very high local concentration of antigens. The conjugation of peptides to the glass surface via primary amines through both terminal NH_2_ groups and free lysines could theoretically block part of a specific binding and generating false negative result. However, during printing conditions N-terminal amines are favoured due to lower pKa values (pKa of the α-amino group = 8.9 and pKa of the ε-amino group of lysine = 10.5). Moreover, this would be compensated during screening analysis as biotinylation of peptides were exclusively performed at the N-terminus. We were able to identify 5 to 40% of the positive binders among the clones screened (Table 2), depending on the phage library. ScFv clones with different binding patterns related to the four possible outcomes were identified. Antibodies targeting carbohydrates generally have a lower affinity than antibodies targeting peptide/protein binders, resulting in the need for a higher concentration of scFv clones to identify positive clones in the capture assay. A higher concentration is needed in the capture assay for the detection of glycopeptide binding (scFv G2-H7) than for peptide binding (scFv H5). The scFv H5 clone targeting the Ku80 protein probably has a higher affinity for the target than in the case of scFv G2-H7. The high number of bis/tris-GalNAc antibodies *versus* the number of mono-GalNAc antibodies in the literature [27–29] provides an indication of the difficulty in generating antibodies against mono-GalNAc epitopes. The repertoire of the phage library generated from immunized mice may favour bis-GalNAc antibodies.

As a proof of concept we were able to identify both peptide and glycopeptide binders demonstrating the possibility of using the combinatory microarray screening assay for screening for affinity maturation of mutated phage libraries, in the search for binders with higher affinity. This gives us a tool for the selection of clones with different characteristics, facilitating the comparison of the outcome of affinity maturation, since an improved scFv G2-H7 clone would give a fluorescence signal in the capture assay after affinity maturation. The flexibility of this combinatory microarray screening assay makes it possible to design the layout after specific requirements, for example increasing the number of peptides tested in the specificity evaluation or reducing the number of peptides to be able to screen increased number of scFv clones.

Although we have demonstrated that scFv clones against small linear antigens (e.g. peptides) can be identified, it is not obvious that they will perform well on relevant biological material. Therefore, the functionality of screened scFv clones was further evaluated on cell lines, using Ku80-positive LnCap cells and Tn-antigen-positive Jurkat cells. We were able to show that both scFv clones were able to bind to biological material and were suitable for flow cytometry analysis.

In conclusion, we were able to efficiently identify new specific scFv binders using defined synthetic peptide/glycopeptide libraries, scFv phage libraries and a newly developed combinatory microarray screening method. The dual antibody/antigen screening and avoidance of a purification step can also reduce time and costs for further development of affinity maturated antibodies.

## Supporting Information

S1 Table Amino acid sequences of additional peptides used in the phage-binding microarray assay. (TIF)

S2 Table MALDI-TOF analysis of peptide BSA conjugates. (DOCX)

S1 Fig The results of screening of scFv clones binding to peptides 1, 6 and 7 in the combinatory microarray screening assay. A) Results of the capture assay for peptide **7**. B) Results of the antigen assay.
(TIF)

S2 Fig Results of screening of scFv clones binding to glycopeptide 9 in the combinatory microarray screening assay. The results of the capture assay were negative, but scFv clones binding to printed glycopeptide 9 could be identified in the second part (antigen assay) of the combinatory microarray screening assay in a four well microarray setup.
(TIF)

## References

[1] Ecker DM, Jones SD, Levine HL. The therapeutic monoclonal antibody market. mAbs. 2015;7(1):9–14. 10.4161/19420862.2015.989042 25529996 PMC4622599

[2] Hoogenboom HR. Selecting and screening recombinant antibody libraries. Nature Biotechnology. 2005;23(9):1105–1116. 10.1038/nbt1126 16151404

[3] Li J, Zhu Z. Research and development of next generation of antibody-based therapeutics. Acta Pharmacol Sin. 2010;31(9):1198–1207. 10.1038/aps.2010.120 20694021 PMC4002304

[4] Ayriss J, Woods T, Bradbury A, Pavlik P. High-Throughput Screening of Single-Chain Antibodies Using Multiplexed Flow Cytometry. Journal of Proteome Research. 2007;6(3):1072–1082. 10.1021/pr0604108 17330944

[5] Schwenk JM, Lindberg J, Sundberg M, Uhlén M, Nilsson P. Determination of Binding Specificities in Highly Multiplexed Bead-based Assays for Antibody Proteomics. Molecular & Cellular Proteomics. 2007;6(1):125–132. 17060675 10.1074/mcp.T600035-MCP200

[6] Kibat J, Schirrmann T, Knape MJ, Helmsing S, Meier D, Hust M, et al Utilisation of antibody microarrays for the selection of specific and informative antibodies from recombinant library binders of unknown quality. New Biotechnology. 2016;33(5, Part A):574–581. 26709003 10.1016/j.nbt.2015.12.003

[7] Seurynck-Servoss SL, Baird CL, Miller KD, Pefaur NB, Gonzalez RM, Apiyo DO, et al Immobilization strategies for single-chain antibody microarrays. PROTEOMICS. 2008;8(11):2199–2210. 10.1002/pmic.200701036 18452230

[8] Abdiche YN, Miles A, Eckman J, Foletti D, Van Blarcom TJ, Yeung YA, et al High-Throughput Epitope Binning Assays on Label-Free Array-Based Biosensors Can Yield Exquisite Epitope Discrimination That Facilitates the Selection of Monoclonal Antibodies with Functional Activity. PLoS ONE. 2014;9(3):e92451 10.1371/journal.pone.0092451 24651868 PMC3961344

[9] Babel I, Barderas R, Peláez-García A, Casal JI. Antibodies on demand: a fast method for the production of human scFvs with minimal amounts of antigen. BMC Biotechnology. 2011;11:61–61. 10.1186/1472-6750-11-61 21635725 PMC3125328

[10] Muller C, Paupert J, Monferran S, Salles B. The Double Life of the Ku Protein: Facing the DNA Breaks and the Extracellular Environment. Cell Cycle. 2005;4(3):438–441. 10.4161/cc.4.3.1565 15738653

[11] Welinder C, Baldetorp B, Blixt O, Grabau D, Jansson B. Primary Breast Cancer Tumours Contain High Amounts of IgA1 Immunoglobulin: An Immunohistochemical Analysis of a Possible Carrier of the Tumour-Associated Tn Antigen. PLoS ONE. 2013;8(4):e61749 10.1371/journal.pone.0061749 23637900 PMC3630176

[12] Ju TA, Rajindra P. | Kudelka Matthew R. | Wang Yingchun | Cummings Richard D. The Cosmc connection to the Tn antigen in cancer. Cancer Biomarkers. 2014;4(1):63–81. 10.3233/CBM-130375 PMC5808877 24643043

[13] Blixt O, Bueti D, Burford B, Allen D, Julien S, Hollingsworth M, et al Autoantibodies to aberrantly glycosylated MUC1 in early stage breast cancer are associated with a better prognosis. Breast Cancer Research: BCR. 2011;13(2):R25–R25. 10.1186/bcr2841 21385452 PMC3219186

[14] Blixt O, Cló E, Nudelman AS, Sørensen KK, Clausen T, Wandall HH, et al A High-Throughput O-Glycopeptide Discovery Platform for Seromic Profiling. Journal of Proteome Research. 2010;9(10):5250–5261. 10.1021/pr1005229 20726594 PMC3001163

[15] Blixt O, Cló E. Synthesis of O-Glycopeptides and Construction of Glycopeptide Microarrays In: Jensen JK, Tofteng Shelton P, Pedersen LS, editors. Peptide Synthesis and Applications. Totowa, NJ: Humana Press; 2013 p. 201–214. 10.1007/978-1-62703-544-6_14 23943488

[16] Zegers N, Gerritse K, Deen C, Boersma W, Claassen E. An improved conjugation method for controlled covalent coupling of synthetic peptides to proteins using glutaraldehyde in a dialysis method. Journal of Immunological Methods. 1990;130(2):195–200. 2115551 10.1016/0022-1759(90)90048-z

[17] Krebber A, Bornhauser S, Burmester J, Honegger A, Willuda J, Bosshard HR, et al Reliable cloning of functional antibody variable domains from hybridomas and spleen cell repertoires employing a reengineered phage display system. Journal of Immunological Methods. 1997;201(1):35–55. 9032408 10.1016/s0022-1759(96)00208-6

[18] Lindner P, Plückthun A. Miniantibodies In: Kontermann R, Dübel S, editors. Antibody Engineering. Springer Lab Manuals: Springer Berlin Heidelberg; 2001 p. 637–647.

[19] Schaefer J, Honegger A, Plückthun A. Construction of scFv Fragments from Hybridoma or Spleen Cells by PCR Assembly In: Kontermann R, Dübel S, editors. Antibody Engineering: Springer Berlin Heidelberg; 2010 p. 21–44.

[20] Shahsavarian MA, Le Minoux D, Matti KM, Kaveri S, Lacroix-Desmazes S, Boquet D, et al Exploitation of rolling circle amplification for the construction of large phage-display antibody libraries. Journal of Immunological Methods. 2014;407:26–34. 10.1016/j.jim.2014.03.015 24681277

[21] Welinder C, Baldetorp B, Borrebaeck C, Fredlund B-M, Jansson B. A new murine IgG1 anti-Tn monoclonal antibody with in vivo anti-tumor activity. Glycobiology. 2011;21(8):1097–1107. 10.1093/glycob/cwr048 21470982

[22] Nakada H, Inoue M, Tanaka N, Numata Y, Kitagawa H, Fukui S, et al Expression of the Tn antigen on T-lymphoid cell line Jurkat. Biochemical and Biophysical Research Communications. 1991;179(2):762–767. 1716888 10.1016/0006-291x(91)91882-d

[23] Turunen L, Takkinen K, Söderlund H, Pulli T. Automated Panning and Screening Procedure on Microplates for Antibody Generation from Phage Display Libraries. Journal of Biomolecular Screening. 2009;14(3):282–293. 10.1177/1087057108330113 19224869

[24] Cai H, Palitzsch B, Hartmann S, Stergiou N, Kunz H, Schmitt E, et al Antibody Induction Directed against the Tumor-Associated MUC4 Glycoprotein. ChemBioChem. 2015;16(6):959–967. 10.1002/cbic.201402689 25755023

[25] Kibat J, Schirrmann T, Knape MJ, Helmsing S, Meier D, Hust M, et al Utilisation of antibody microarrays for the selection of specific and informative antibodies from recombinant library binders of unknown quality. New Biotechnology. 10.1016/j.nbt.2015.12.003 26709003

[26] Angenendt P, Wilde J, Kijanka G, Baars S, Cahill DJ, Kreutzberger J, et al Seeing Better through a MIST: Evaluation of Monoclonal Recombinant Antibody Fragments on Microarrays. Analytical Chemistry. 2004;76(10):2916–2921. 10.1021/ac035357a 15144205

[27] Yuasa N, Ogawa H, Koizumi T, Tsukamoto K, Matsumoto-Takasaki A, Asanuma H, et al Construction and expression of anti-Tn-antigen-specific single-chain antibody genes from hybridoma producing MLS128 monoclonal antibody. Journal of Biochemistry. 2012;151(4):371–381. 10.1093/jb/mvs007 22318767

[28] Loureiro LR, Carrascal MA, Barbas A, Ramalho JS, Novo C, Delannoy P, et al Challenges in Antibody Development against Tn and Sialyl-Tn Antigens. Biomolecules. 2015;5(3):1783–1809. 10.3390/biom5031783 26270678 PMC4598775

[29] Ando H, Matsushita T, Wakitani M, Sato T, Kodama-Nishida S, Shibata K, et al Mouse-Human Chimeric Anti-Tn IgG1 Induced Anti-tumor Activity against Jurkat Cells in Vitro and in Vivo. Biological and Pharmaceutical Bulletin. 2008;31(9):1739–1744. 18758069 10.1248/bpb.31.1739

