# Supplementary figures and images for: A Combinatory Antibody–Antigen Microarray Assay for High-Content Screening of Single-Chain Fragment Variable Clones from Recombinant Libraries
Source: PLoS One. 2016 Dec 21;11(12):e0168761. doi: 10.1371/journal.pone.0168761 (PMC5176327; doi:10.1371/journal.pone.0168761)

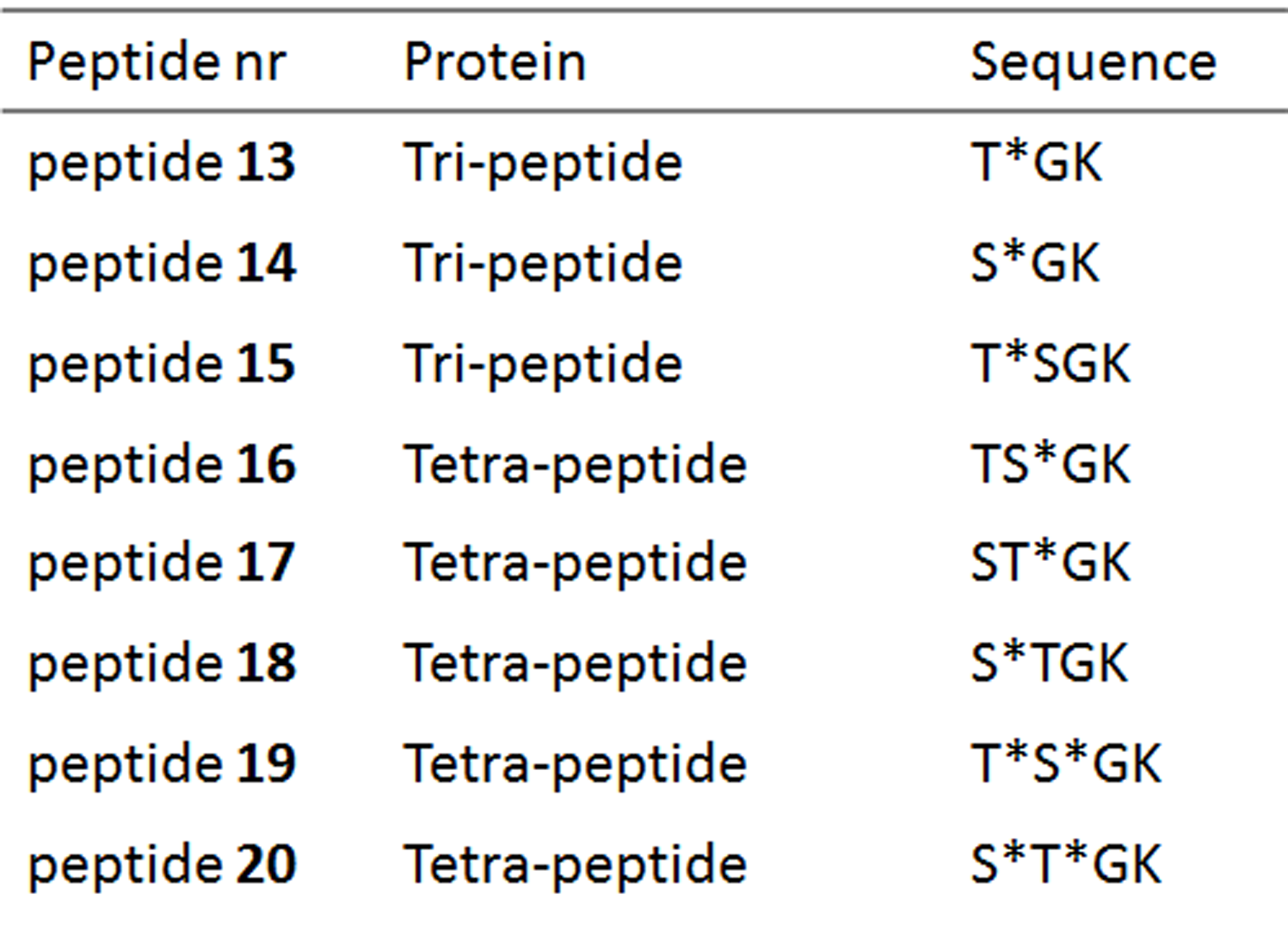

Supplement: S1 Table — (TIF) [file pone.0168761.s001.tif]

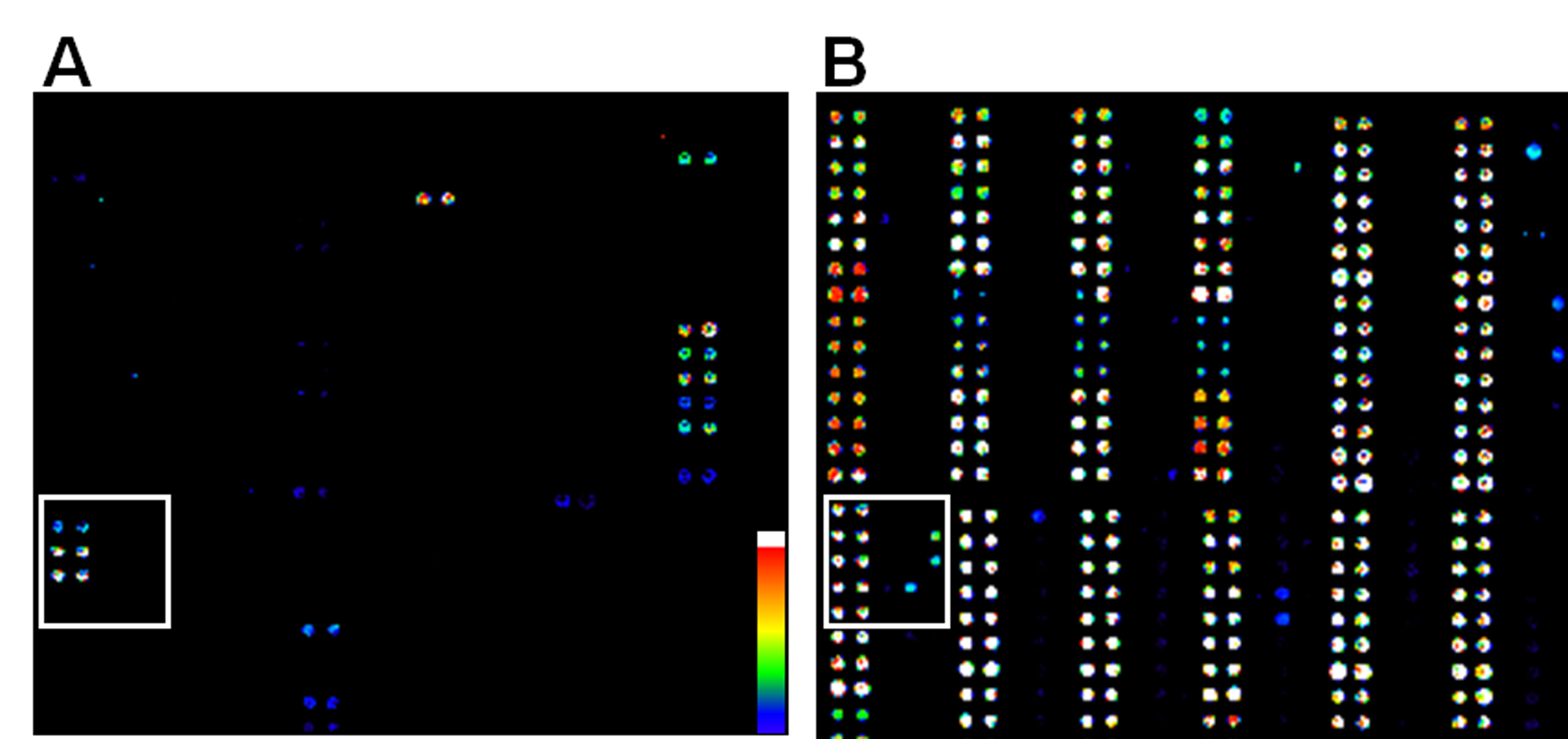

Supplement: S1 Fig — A) Results of the capture assay for peptide 7. B) Results of the antigen assay. (TIF) [file pone.0168761.s003.tif]

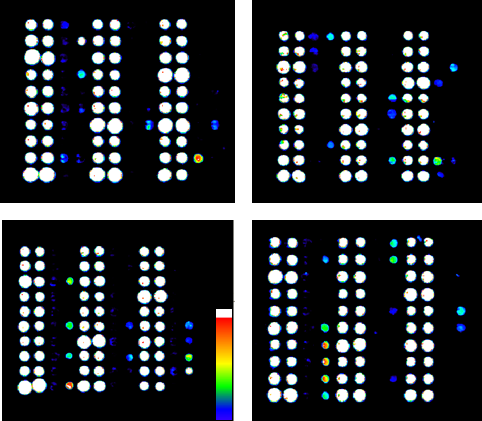

Supplement: S2 Fig — The results of the capture assay were negative, but scFv clones binding to printed glycopeptide 9 could be identified in the second part (antigen assay) of the combinatory microarray screening assay in a four well microarray setup. (TIF) [file pone.0168761.s004.tif]
