# Supplementary material for: A Combinatory Antibody–Antigen Microarray Assay for High-Content Screening of Single-Chain Fragment Variable Clones from Recombinant Libraries
Source: PLoS One. 2016 Dec 21;11(12):e0168761. doi: 10.1371/journal.pone.0168761 (PMC5176327; doi:10.1371/journal.pone.0168761)

|  |  | |  | |  |  | | |  | | |  | |  | |  |  |
| --- | --- | --- | --- | --- | --- | --- | --- | --- | --- | --- | --- | --- | --- | --- | --- | --- | --- |
| **Peptide nr.** | **Mw Maldi** | | **Mw-BSA** | | **peptide incorporation** | | |  |  |  |  |  |  |  |  |  |  |
| Peptide 1 | 80000 | | 13500 | | 6,242976651 | | |  |  |  |  |  |  |  |  |  |  |
| Peptide 2 | 77700 | | 11200 | | 6,983370848 | | |  | | |  | |  | |  |  |  |
| Peptide 6 | 78000 | | 11500 | | 4,180161318 | | |  | | |  | |  | |  |  |  |
| Peptide 7 | 78200 | | 11700 | | 4,448736863 | | |  | | |  | |  | |  |  |  |
| Peptide 1 | |  | |  | | |  | | |  |  |  |  |  |  |  |  |


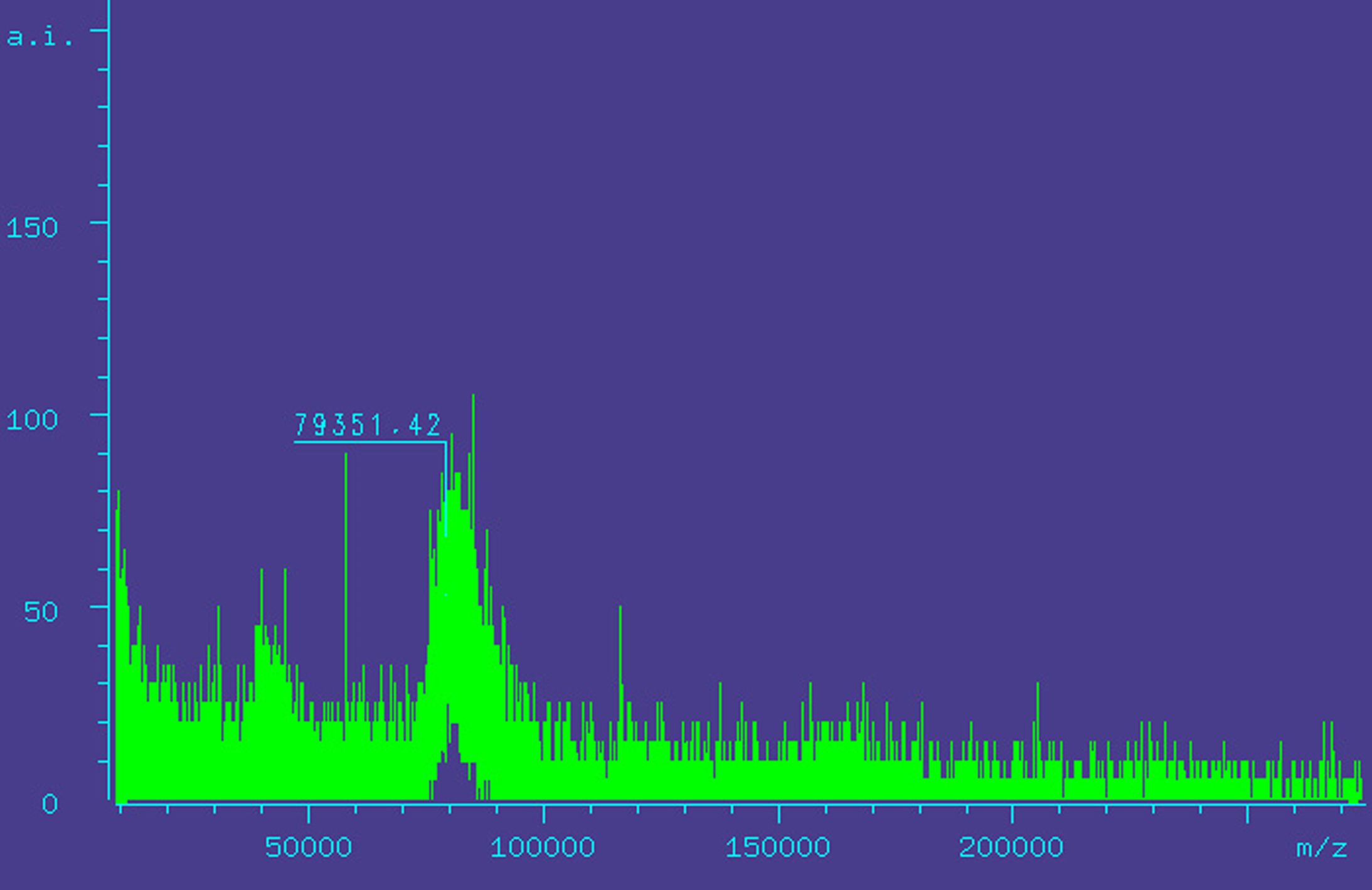


Peptide 2


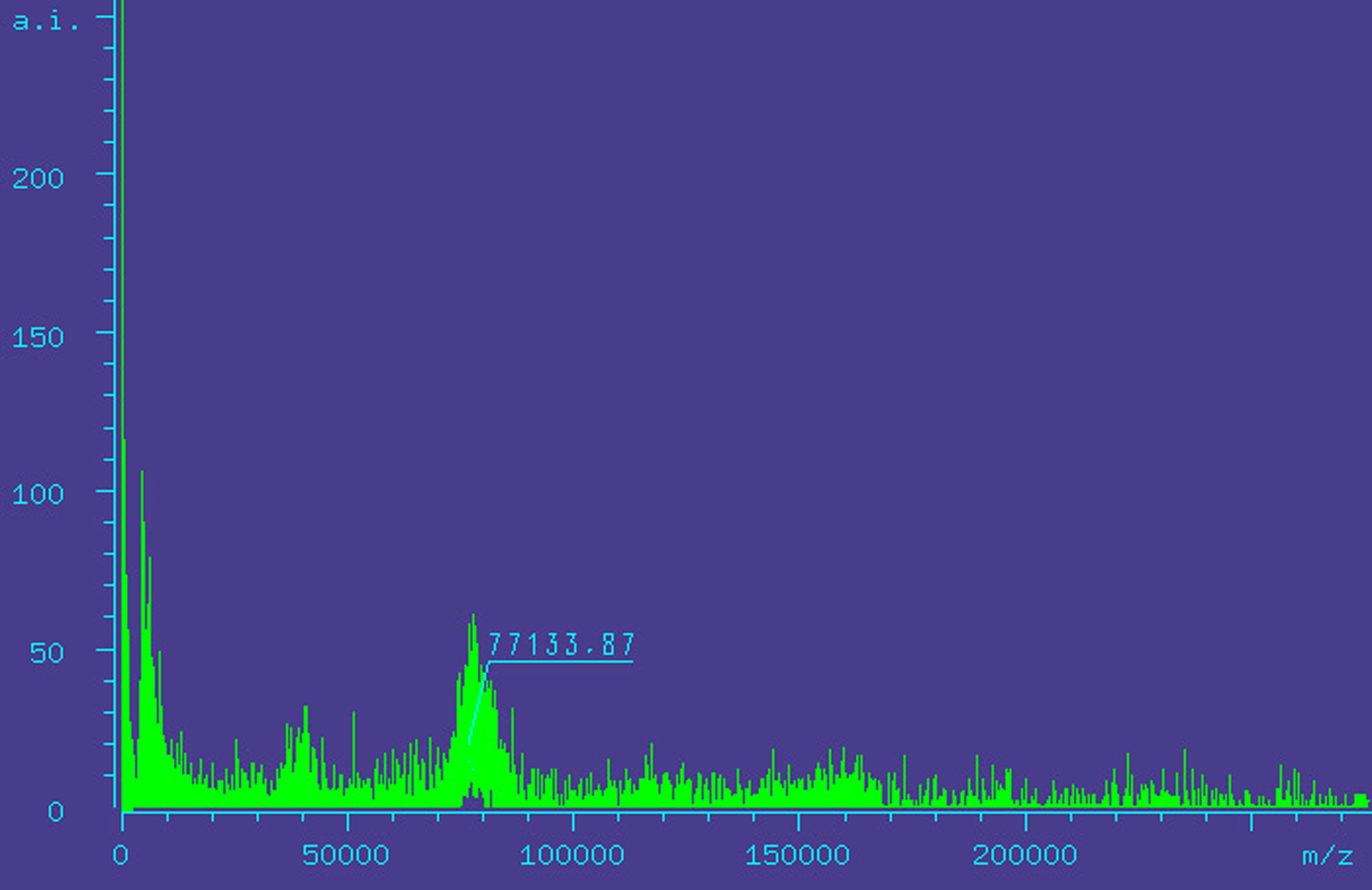


Peptide 6


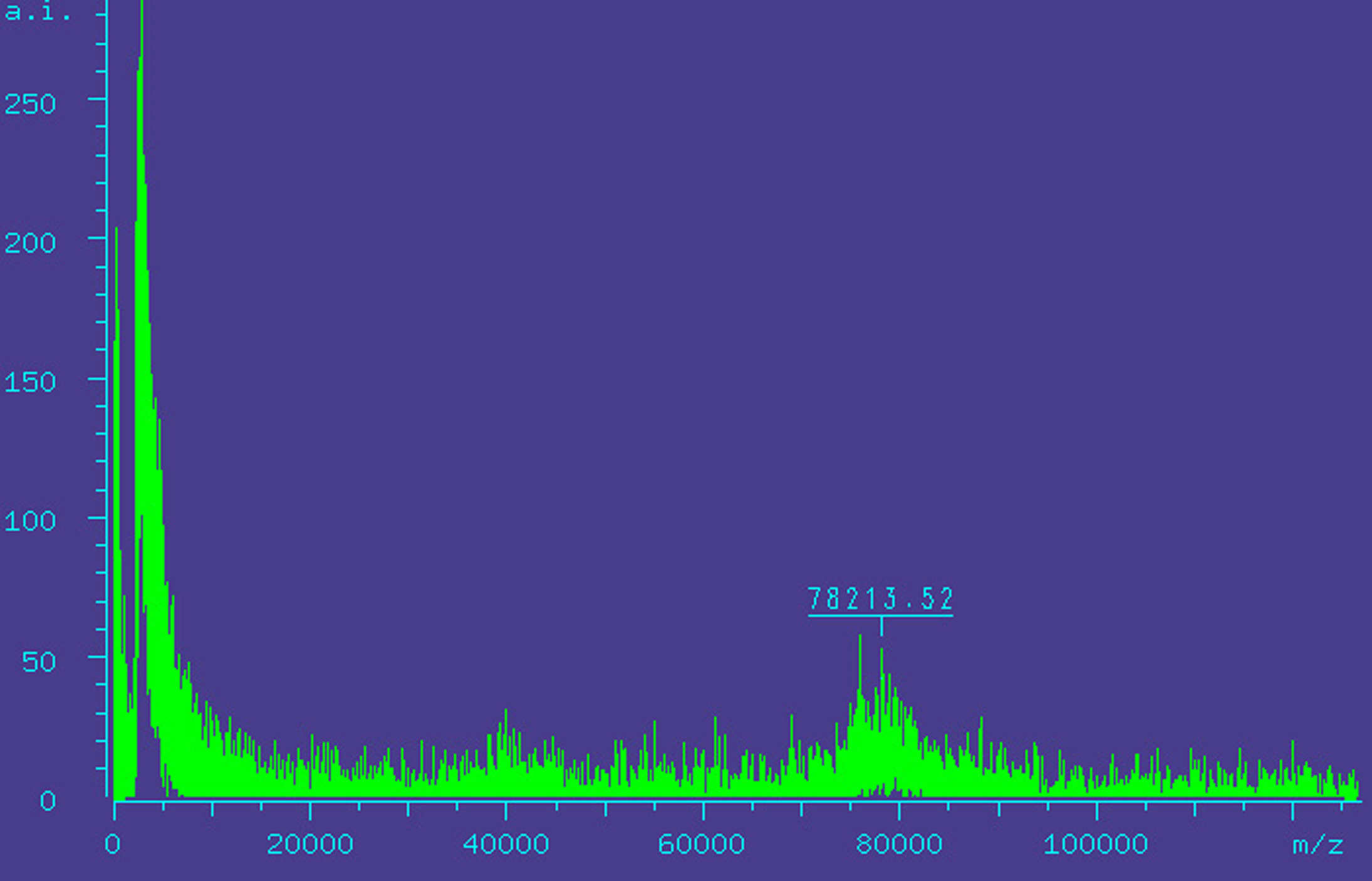


Peptide 7


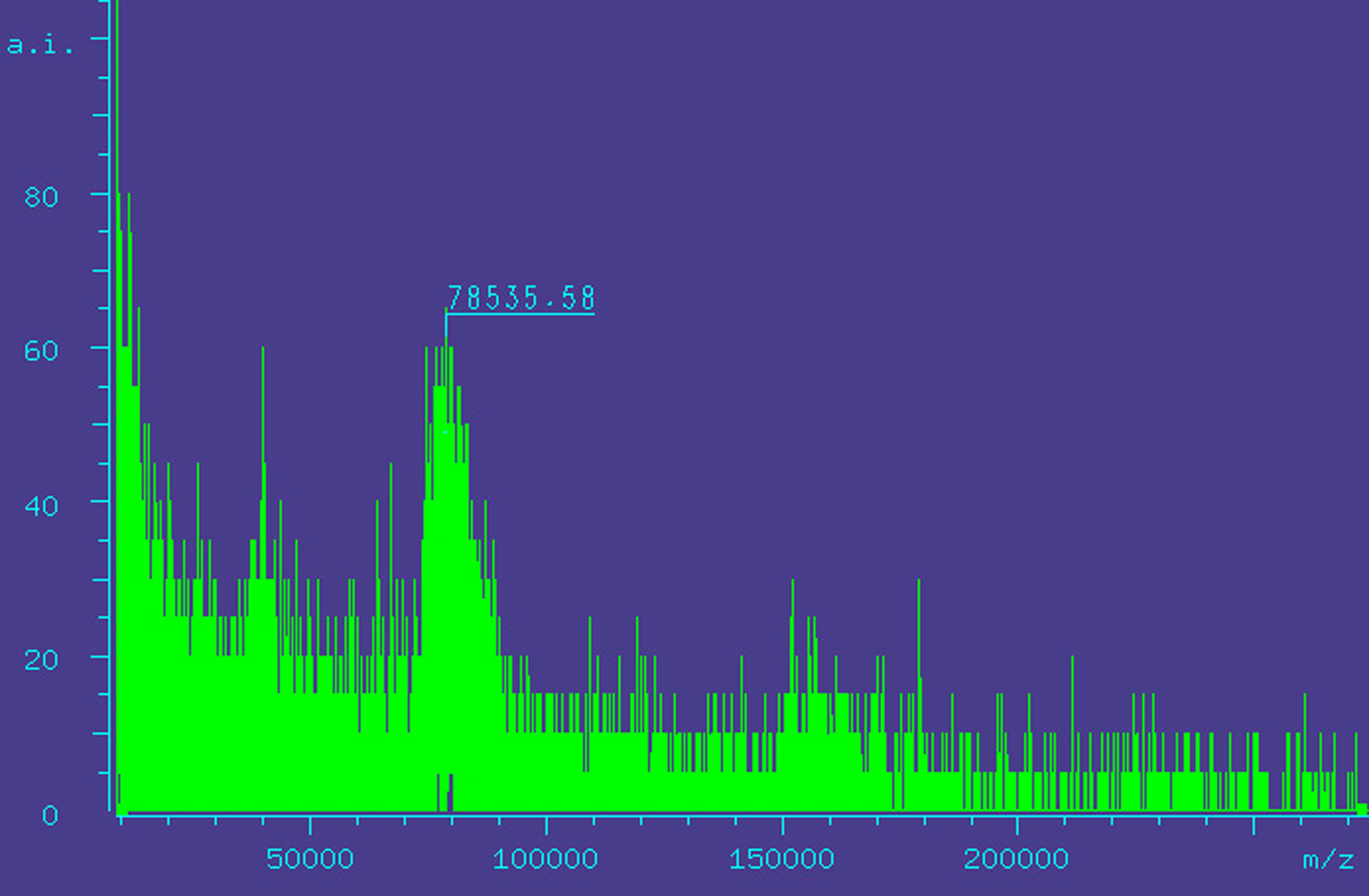

Supplement: S2 Table — (DOCX) [file pone.0168761.s002.docx]
